# High Hip Center Technique in Total Hip Arthroplasty for Crowe Type II–III Developmental Dysplasia: Results of Midterm Follow‐up

**DOI:** 10.1111/os.12756

**Published:** 2020-08-09

**Authors:** Junmin Shen, Jingyang Sun, Haiyang Ma, Yinqiao Du, Tiejian Li, Yonggang Zhou

**Affiliations:** ^1^ Medical School of Chinese People's Liberation Army Beijing China; ^2^ Department of Orthopedics, The First Medical Centre Chinese People's Liberation Army General Hospital Beijing China

**Keywords:** Crowe Type II‐III, Developmental Dysplasia of the Hip, High Hip Center, Total Hip Arthroplasty

## Abstract

**Objectives:**

We aimed to show the utility of high hip center technique used in patients with Crowe II–III developmental dysplasia of the hip at the midterm follow‐up and evaluated the clinical and radiographic results between different heights of hip center.

**Methods:**

From December 2003 to November 2013, we retrospectively evaluated 69 patients (85 hips) with Crowe II–III dysplasia who underwent a high hip center cementless total hip arthroplasty. The patients were divided into two groups according to the height of hip center, respectively group A (≥ 22 mm and < 28 mm) and group B (≥28 mm). The survivorship outcomes and radiographic and clinical results, including the vertical and horizontal distances of hip center, femoral offset, abductor lever arm, cup inclination, leg length discrepancy, Trendelenburg sign, and limp were evaluated.

**Results:**

The mean follow‐up time was 8.9 ± 1.8 years. The mean location of the hip center from the inter‐teardrop was 25.1 ± 1.6 mm vertically and 30.0 ± 3.8 mm horizontally in group A, and 33.1 ± 4.8 mm vertically and 31.4 ± 6.1 mm horizontally in group B. Eleven hips of group B showed a lateralization over 10 mm, and the same was shown in one hip in group A (*P* = 0.012). There were no statistically significant differences between two groups in postoperative femoral offset, abductor lever arm, leg length discrepancy and cup inclination. At the final follow up, the mean WOMAC and Harris hip score were significantly improved in both groups. Of the 85 hips, four hips in group A and three hips in group B showed a positive Trendelenburg sign. Additionally, four patients in group A and two patients in group B presented with a limp. No significant differences were shown regarding the Harris hip score, WOMAC score, Trendelenburg sign, and limp between two groups. One hip of group A was revised by reason of dislocation at 8.3 years after surgery. One hip of group B was diagnosed with osteolysis and underwent a revision at 8.1 years after surgery. The Kaplan–Meier implants survivorship rates at the final follow‐up for all‐causes revisions in group A and group B were similar (96.7% [95% confidence interval, 90.5%–100%] and 96.2% [95% confidence interval, 89.0%–100%], respectively).

**Conclusions:**

The high hip center technique is a valuable alternative to achieve excellent midterm results for Crowe II–III developmental dysplasia of the hip. Further, between the groups with differing degrees of HHC, there were no significant differences in outcomes or survivorship in our study.

## Graphical abstract

High hip center technique performing well in THA for Crowe II‐III DDH

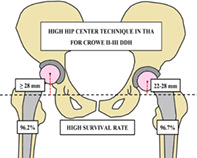

## Introduction

Total hip arthroplasty (THA) in developmental dysplasia of the hip (DDH) presents technical challenges due to complex acetabular and femoral deformities which can be classified by the Crowe classification.^1^, ^2^ For Crowe type II–III DDH which always encompasses segmental or complete absence of a superolateral rim, the issue of acetabular reconstruction should be priority. Previously, aiming to restore the normal hip biomechanical mechanism, most studies concurred that the true acetabulum was the optimal location for the cup.^3^ To achieve anatomical placement of the cup, augmentation by structural bone graft to supplement bone insufficiency was commonly required in spite of making the procedure complicated and time‐consuming.^4^ Nevertheless, high failure rates of bone graft have been revealed in the literature by reason of bone graft resorption and collapse.^5^

In 1991, Russotti and Harris^3^ proposed proximal placement of the acetabular component in revision THA, commonly called “high hip center (HHC).” The advantages of HHC include optimum bone ingrowth with greater bone‐implant contact and simplification of the operation. For patients with Crowe II–II DDH, HHC technique has been discussed as a potential alternative option to address the problem of acetabular deficiency. However, previous studies have shown superolateral placement could result in accelerated polyethylene (PE) wear, decreased abductor moment arm and component loosening.^6^ In contrast, more recent clinical studies have demonstrated promising results of this technique. Kaneuji *et al*.^7^ reported no cup loosening in 30 hips (29 patients) using HHC technique for a mean of 15.2 years after surgery. Nawabi *et al*.^8^ showed no difference in survivorship, wear rates and hip scores between the HHC group and the control group. Even so, high placement of the cup is still controversial and more mid‐ to long‐term follow‐up studies are required.

When considering HHC THA, Schutzer and Harris^9^ defined 28 mm above the inter‐teardrop line as a high hip center, which was at least two times higher than the normal level. In contrast, Fukui *et al*.^10^ defined 22 mm above the inter‐teardrop line as the high rotation center for Japanese people. Therefore, different authors have suggested different definitions for HHC, but there is no research comparing different heights of HHC.

The aim of this study was: (i) to assess the utility of HHC technique used in patients with Crowe II–III DDH at midterm follow‐up; (ii) to evaluate the clinical and radiographic outcomes between different heights of HHC.

## Patients and Methods

### *Inclusion and Exclusion Criteria*

The inclusion criteria were: (i) adult patients with Crowe type II or III DDH; (ii) patients who received cementless THA from a single surgeon in our institution between December 2003 and November 2013; (iii) the acetabular cup was placed at the high hip center; (iv) patients divided into two groups based on the height of hip center; (v) outcome measures included the cup position, femoral offset (FO), abductor lever arm (ALA), cup inclination, leg length discrepancy (LLD), Trendelenburg sign, postoperative limp, Harris hip score (HHS), WOMAC index, and survivorship; and (vi) retrospective study. The exclusion criteria included: (i) revised cases; and (ii) patients with histories of neuromuscular disease.

### *Patients*

After obtaining institutional review board approval, we performed a retrospective analysis of a case series. From our departmental database, we identified 76 patients diagnosed with Crowe II–III dysplasia with the acetabular cup placed at the high hip center, of which the threshold was defined as 22 mm above the inter‐teardrop line.^10^ One patient died of an unrelated cause to the procedure at 8 years after surgery and four patients were lost to follow‐up. Two patients refused to participate for questionnaires and clinical examination. Therefore, 69 patients (85 hips) were ultimately available for this study.

According to the Crowe classification, 49 hips were categorized as type II and 36 hips were categorized as type III. Eleven patients had a history of previous surgeries: open reduction in one case, femoral derotational osteotomy in two cases, pelvic osteotomy in three cases, and hip shelf procedure in five cases.

### *Groups according to the height of hip center*

The patients were divided into two groups according to the height of hip center. In group A, which consisted of 39 hips, the hip center was located at a vertical distance of ≥22 mm and<28 mm from the inter‐teardrop line, when the hip center of group B which consisted of 46 hips was ≥28 mm (Table 1).

**TABLE 1:** Demographics of the Patients

Demographic	Group A	Group B	*P* value
Number of hips (patients)	39 (31)	46 (38)	
Age (years) ^*^	46.5 ± 12.6	46.4 ± 11.0	0.959
Gender (n)			0.665
Male	6 (19%)	9 (24%)	
Female	25 (81%)	29 (76%)	
Height (cm) ^*^	161.1 ± 8.4	161.8 ± 9.0	0.723
BMI (kg/m^2^) ^*^	23.7 ± 3.3	24.5 ± 4.0	0.333
Side (n)			0.323
Right	17 (44%)	25 (54%)	
Left	22 (56%)	21 (46%)	
Crowe classification(hips)			<0.001
Type II	32 (82%)	17 (37%)	
Type III	7 (18%)	29 (63%)	

BMI, body mass index.

* The values are given as the mean and standard deviation.

### *Surgical technique*

#### *Anesthesia and Position*

All procedures were performed with the patient in the lateral decubitus position and under general anesthesia.

#### *Approach and Exposure*

All operations were performed using a modified Kocher‐Langenbeck posterolateral approach. The fascia was divided along the line of skin incision and the gluteus maximus was split in the direction of its fibers. The short external rotators were divided as close to their insertion on the femur. Subsequently, the hip joint was posteriorly dislocated.

#### *Resection and preparation*

Using an oscillating saw, the femoral head was resected based on the distance from the lesser trochanter by preoperative templating. The acetabulum was reamed medially and proximally.

#### *Placement of prosthesis*

The adjustment of cup orientation and intentional medial placement were adopted, aiming to achieve a bone‐cup surface contact not inferior to 70%. Partial uncoverage of the superolateral rim was deemed acceptable when good stability was achieved. No superior acetabular grafts or spongioplasty were used in all operations. In some case, a larger size stem was used to elevate the position of the stem in the femoral canal with different head/neck lengths, aiming to restore the proper tension of the gluteus medius and correct limb‐length discrepancy. The detailed information of acetabular and femoral components and types of bearing were shown in Table 2.

**TABLE 2:** Specific designs of acetabular and femoral components and types of bearing used in all patients

	Group A	Group B
Median cup size (mm) (IQR)	50 (50, 52)	50 (48, 52)
Acetabular component		
Betacup (Link, Hamburg, Germany)	20 (51.3%)	24 (52.2%)
Duraloc (DePuy, Warsaw, IN, USA)	11 (28.2%)	9 (19.5%)
Pinnacle (DePuy, Warsaw, IN, USA)	7 (17.9%)	13 (28.3%)
Trident (Stryker, Mahwah, NJ, USA)	1 (2.6%)	‐
Femoral stem		
Corail (DePuy, Warsaw, IN, USA)	32 (82.0%)	31 (67.4%)
S‐ROM (DePuy, Warsaw, IN, USA)	5 (12.8%)	12 (26.1%)
Ribbed (Link, Hamburg, Germany)	1 (2.6%)	2 (4.3%)
LCU (Link, Hamburg, Germany)	‐	1 (2.2%)
Accolade (Stryker, Mahwah, NJ, USA)	1 (2.6%)	‐
Bearing type		
COC	36 (92.3%)	42 (91.3%)
COP	2 (5.1%)	2 (4.35%)
MOP	1 (2.6%)	2 (4.35%)

IQR, interquartile range; COC, ceramic on ceramic; COP, ceramic on polyethylene; MOP, metal on polyethylene.

#### *Postoperative Reconstruction*

All patients received antithrombotic prophylaxis using low‐molecular‐weight heparin postoperatively. We advised the patient to load the surgically treated leg using two crutches for 6 weeks.

### *Radiographic Evaluation*

Radiological assessment based on anteroposterior (AP) radiograph of the pelvis was undertaken for all patients preoperatively, postoperatively, and at last follow up.

#### *Osteolysis, Radiolucent Line and Loosening*

Osteolysis was defined as circular or oval areas of distinct bone loss. The location of radiolucent lines with a width of over 1 mm at the component‐bone interface was described according to DeLee and Charnley.^11^ The cup was considered loosened in presence of a change of more than 3 mm of migration or at least 4° in the angle of abduction.^12^

#### *Cup Position and Inclination*

**Fig 1:**
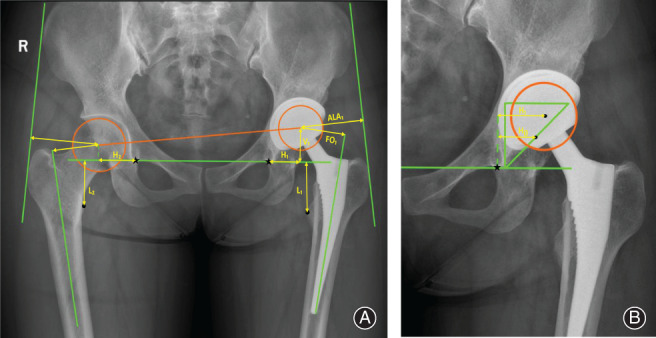
(A) Diagram for radiographic measurement of unilateral HHC; (B) Ranawat triangle was drawn to define the anatomic hip center of bilateral HHC. The star represents teardrop and the dot represents the apex of the lesser trochanter. V: vertical distance; H: horizontal distance; L: leg length; FO: femoral offset; ALA: abductor lever arm; △H = H_2_‐H_1_ (unilateral HHC) or H_0_‐H_1_ (bilateral HHC), positive indicates medialization and negative indicates lateralization.

The position of the cup was defined as the vertical and horizontal distances of the center of rotation in relation to the acetabular teardrop. The cup inclination was defined as the abduction angle, formed by the inter‐teardrop line and the connecting line to the edges of the rim of the cup (Fig. 1).

#### *Medialization*

Medialization was measured as the difference in the horizontal distance of the center of rotation in relation to the teardrop between the elevated hip and contralateral hips. In unilateral HHC, medialization was measured by contrast with the contralateral hip. In bilateral HHC, the Ranawat triangle was drawn to define the correct anatomic hip center to calculate the medialization^13^ (Fig. 1).

#### *Leg Length Discrepancy*

The LLD was measured as the difference in distance between the tip of the lesser trochanter and the inter‐teardrop line, connecting the caudal margins of the teardrop on the two sides (Fig. 1).

#### *Femoral Offset and Abductor Lever Arm*

The FO was defined as the length from the center of rotation to the perpendicular line drawn under the central axis of the femur. The ALA was measured from the femoral head to the line joining the lateral part of the greater trochanter to the anterosuperior iliac crest (Fig. 1).

### *Clinical Assessment*

We clinically evaluated each patient with the Harris Hip Score, WOMAC score, Trendelenburg sign, and postoperative limp.

**TABLE 4:** Clinical evaluation

Parameters	Group A	Group B	*P* value
Preoperative HHS^*^	53.5 ± 8.0	51.1 ± 8.6	0.199
HHS at last follow‐up^*^	94.0 ± 4.1	92.8 ± 4.5	0.187
Preoperative WOMAC^*^	55.5 ± 6.0	53.9 ± 9.2	0.340
WOMAC at last follow‐up^*^	92.4 ± 6.8	91.6 ± 8.5	0.640
Positive Trendelenburg sign (hips)	4 (10.3%)	3 (6.5%)	0.819
Postoperative limp (patients)	4 (12.9%)	2 (5.3%)	0.526

* The values are given as the mean and standard deviation.

#### *Harris Hip Score (HHS)*

The HHS was used to evaluate postoperative recovery of hip function. The HHS score system mainly includes four aspects as pain, function, absence of deformity, and range of motion. The score standard has a maximum of 100 points (best possible outcome). A total score < 70 is considered a poor score, 70–80 is fair, 80–90 is good, and 90–100 is excellent.

#### *Western Ontario and McMaster Universities Arthritis (WOMAC) Index*

The WOMAC index is used to assess patients with osteoarthritis of the hip. It can be used to monitor the postoperative recovery of hip function. This system mainly includes 24 parameters and the score standard has a maximum of 96 points. In this study, to improve interpretability, the scores for WOMAC were transformed, so that a score of 100 indicated the best state of health and a score of 0 indicated the worst state.^14^

#### *Trendelenburg Sign and Postoperative limp*

Trendelenburg sign was used to assess the muscle strength of gluteus medius. A negative Trendelenburg sign was defined as that when the examiner asked patients to lift one leg off the ground with the hip flexed, the pelvis on the non‐weight‐bearing side could be elevated high and the patients could maintain this position for at least 5 seconds. Any visual evidence of a lateral imbalance in the pelvic movement during gait was scored as a limp.^15^

### *Statistical Analysis*

Continuous variables such as demographics, radiographic measurements, and clinical scores were expressed as mean and standard deviation. The cup size was expressed as median and interquartile range. The categorical variables were assessed by chi‐squared test. Differences in mean parameter values between groups were assessed by Student's t‐test. The end point for survival was defined as revision for any reason. Kaplan–Meier analysis was performed to determine the probability of survivorship in both groups. The equality of the survival distributions between two groups was compared by log‐rank test. Significance was set at *P <* 0.05. All analyses were performed using SPSS Version 25.0 software (IBM, Armonk, NY, USA).

## Results

### *Follow‐up and General Results*

The mean follow‐up time was 8.9 ± 1.8 years (range from 6.0 to 14.1) for all 69 patients and the follow‐up time in group A and group B were 9.5 ± 1.6 years and 8.4 ± 1.7 years, respectively (*P* = 0.007). In group A, the mean duration of surgery was 1.9 ± 0.4 hours, and the mean intraoperative blood loss was 404 ± 222 ml. In group B, the mean duration of the procedure was 2.1 ± 0.7 hours, and the mean intraoperative blood loss was 441 ± 318 ml. No significant difference was shown in duration of surgery (*P* = 0.173) and intraoperative blood loss (*P* = 0.544) between two groups.

### *Radiographic Results*

#### *Osteolysis, Radiolucent Line, and Loosening*

At the final follow‐up, slight osteolysis was observed in two hips in DeLee and Charnley zone 1. These two hips were all from group A. No loosening or progressive radiolucency adjacent to the acetabular and femoral component was observed.

#### *Cup Position and Inclination*

The mean location of the cup from the inter‐teardrop was 25.1 ± 1.6 mm vertically and 30.0 ± 3.8 mm horizontally in group A, and 33.1 ± 4.8 mm vertically and 31.4 ± 6.1 mm horizontally in group B. The mean cup inclination between group A and group B were 41.1 ± 5.2 ° and 41.2 ± 7.2 °, respectively (*P* = 0.955). Therefore, except for the height of cup, there was no significant difference in horizontal position (*P* = 0.212) and inclination between the groups (Table 3).

**TABLE 3:** Postoperative radiographic evaluation

Evaluation parameter	Group A^*^	Group B^*^	*P* value
Vertical distance (mm)	25.1 ± 1.6	33.1 ± 4.8	
Horizontal distance (mm)	30.0 ± 3.8	31.4 ± 6.1	0.212
Femoral offset (mm)	32.9 ± 5.8	32.2 ± 8.0	0.636
Abductor lever arm (mm)	54.0 ± 6.7	52.1 ± 7.5	0.233
Leg length discrepancy (mm)	5.0 ± 2.9	5.5 ± 5.7	0.628
Cup inclination (degree)	41.1 ± 5.2	41.2 ± 7.2	0.955

* The values are given as the mean and standard deviation.

#### *Medialization*

**Fig 2:**
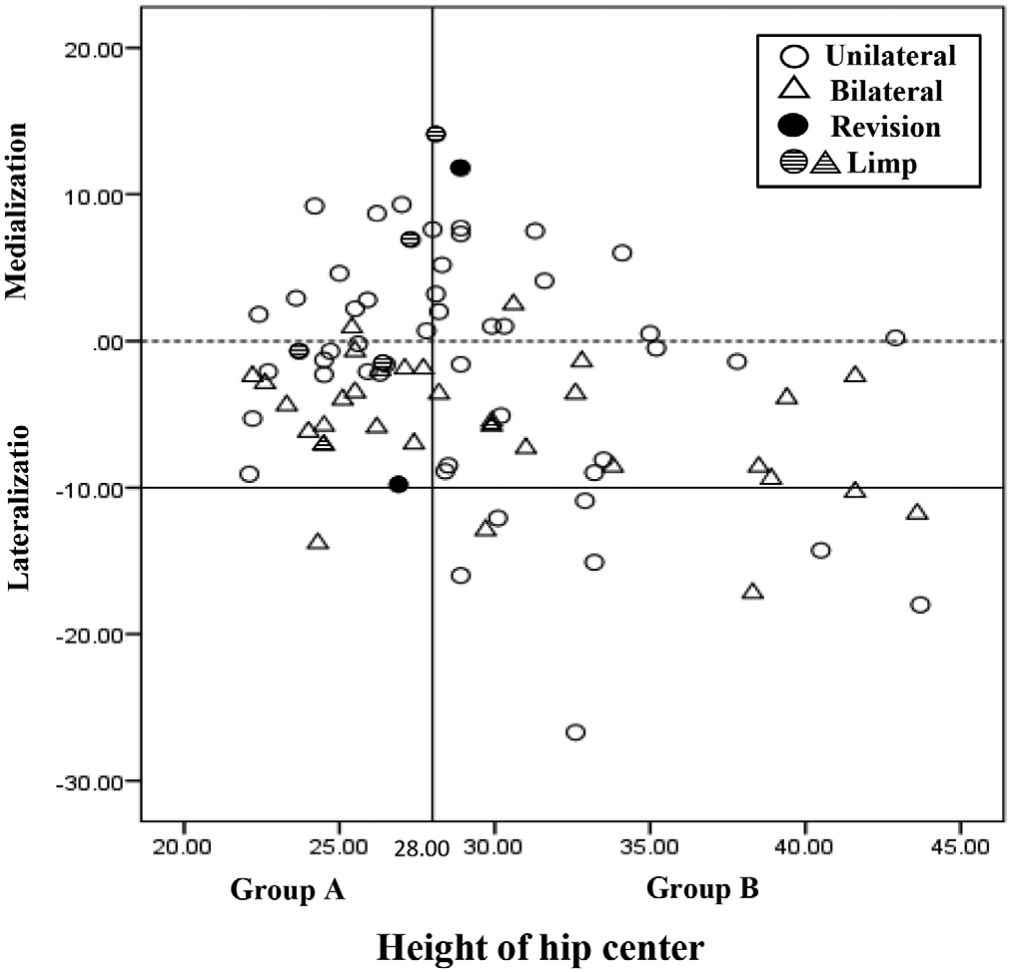
Scatter‐gram of medialization or lateralization in the group A and group B.

Scatter diagram demonstrates the distribution of hip center relative to the anatomic center (Fig. 2). In group B, 11 hips showed a lateralization over 10 mm, as did one hip in the group A (*P* = 0.012). For Crowe III hips, eight of 36 hips were reconstructed more than 10 mm lateral to the anatomic center, and for Crowe II hips, four of 49 hips were reconstructed in more than 10 mm lateral position (*P* = 0.066). Furthermore, between unilateral (53 hips) and bilateral (32 hips) HHC, there is no significant difference of lateralization ≥10 mm (*P* = 0.756).

#### *Leg Length Discrepancy, Femoral Offset, and Abductor Lever Arm*

In group A, the LLD, FO, and ALA were 5.0 ± 2.9 mm, 32.9 ± 5.8 mm and 54.0 ± 6.7 mm, respectively. In group B, those were 5.5 ± 5.7 mm, 32.2 ± 8.0 mm and 52.1 ± 7.5 mm, respectively. No significant difference was observed regarding LLD (*P* = 0.628), FO (*P* = 0.636), and ALA (*P* = 0.233) between the two groups (Table 3).

### *Clinical results*

#### *HHS and WOMAC*

The HHS and WOMAC at the time of follow‐up were significantly improved in both group A (*P* < 0.001) and group B (*P* < 0.001). In group A, the mean HHS improved from 53.5 ± 8.0 points to 94.0 ± 4.1 points and the mean WOMAC improved from 55.5 ± 6.0 points to 92.4 ± 6.8 points. In group B, the mean HHS improved from 51.1 ± 8.6 points to 92.8 ± 4.5 points and the mean WOMAC improved from 53.9 ± 9.2 points to 91.6 ± 8.5 points. There was no significant difference regarding HHS (*P* = 0.187) and WOMAC (*P* = 0.640) in the final follow‐up between the groups (Table 4).

#### *Trendelenburg Sign and Postoperative Limp*

Of the 85 hips, four hips in group A and three hips in group B showed a positive Trendelenburg sign (*P* = 0.819). Additionally, four patients in group A and two patients in group B presented with a limp (*P* = 0.526). No significant difference was shown regarding positive Trendelenburg sign and limp between two groups (Table 4).

#### *Revisions and Kaplan–Meier Survival Rate*

**Fig 3:**
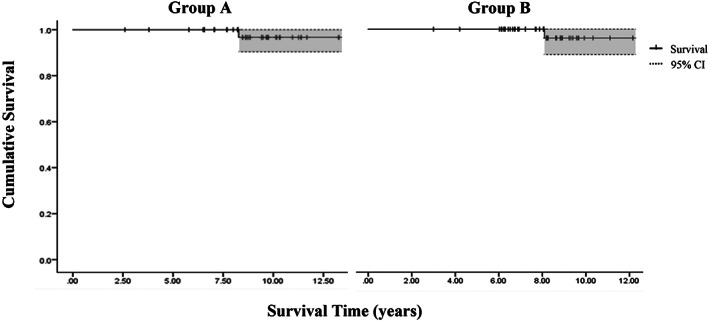
The Kaplan–Meier survival curve with revision for any reason as the end point for group A and group B was shown. CI, confidence interval.

**Fig 4:**
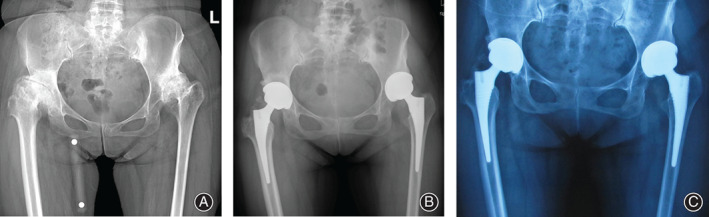
Preoperative (A) anterior–posterior X‐ray highlighted a bilateral DDH (right hip as Crowe II and left hip as Crowe III) in a 47‐year‐old female patient (Crowe index: 0.13 in right hip and 0.16 in left hip). The postoperative (B) anteroposterior radiographic measurement showed that the height of rotation center was 29.7 mm in right hip and 38.5 mm in left hip. At the final follow‐up, the radiographic evaluation (C) after 8.8 years showed no osteolysis and radiolucent line.

Of the 85 hips, two hips (2.4%) required revision during the follow‐up period. One hip of group A was revised by reason of dislocation at 8.3 years after surgery. The other one hip of group B, which utilized a metal on conventional polyethylene bearing at the primary THA, was diagnosed with osteolysis and underwent a revision at 8.1 years after surgery. With revision for any reason as the end point, the Kaplan–Meier survival rates at last follow‐up were similar (*P* = 0.805) in both groups, 96.7% (95%CI, 90.5%–100%) in group A and 96.2% (95%CI, 89.0%–100%) in group B (Figs 3 and 4).

## Discussion

### *High Hip Center Technique and Bone Graft*

The reconstruction of the acetabulum in patients with Crowe II–III DDH is a demanding procedure for orthopaedic surgeons. Most surgeons find it technically difficult to achieve acceptable cup coverage at the anatomical acetabulum on account of superolateral bone deficiency.^16^ Therefore, femoral head structural autograft was usually utilized at the superolateral rim to provide additional support.^17^ However, other authors have proposed the instability of cemented acetabular component with bulk bone grafts.^18^ Though some excellent results were reported in cementless THA with autograft,^19^, ^20^ this procedure still could be correlated with longer duration of surgery and increased blood loss. Because the posterosuperior bone above the native acetabulum is almost intact, the acetabular cup can be placed at high hip center to optimize host bone‐implant contact.^21^ In this study, we aimed to assess the utility of HHC technique used in patients with Crowe II–III DDH and evaluated the clinical and radiographic results between different heights of hip center.

### *The Importance of Medial Placement of the Cup*

Early results have shown superior placement and especially lateralization of the cemented acetabular cup resulted in a high rate of loosening.^6^ In addition, in the cementless THA, aseptic loosening also occurred in long‐term follow‐up. Watts *et al*.^22^ reviewed 88 primary cementless THA at a mean follow‐up of 10 years and found a higher incidence of aseptic loosening and cup revision with superolateral placement of the cup, which was described as more than 10 mm superior and 10 mm lateral to the approximate femoral head center. To avoid this situation, the acetabular component was placed medially adjacent to medial wall during operation in our study. Medialization not only prevented an increase joint reaction force, but biomechanically relieved the burden of abductor muscle which was mostly dysfunctional preoperatively due to chronically shortened condition and subsequent atrophy. In our study, the mean horizontal distance of the center of rotation which was 30.0 mm in group A and 31.4 mm in group B was comparable to the results described by Flecher *et al*.^23^ (horizontal distance was 30.4 mm when vertical distance was 23.4 mm), Fukui *et al*.^10^ (horizontal distance was 28.9 mm when vertical distance was 28 mm), and Galea *et al*.^24^ (horizontal distance was 31.6 mm when vertical distance was 30.9 mm). However, referring to the anatomical center, only 73 (85.9%) acetabular cups attained the objective of medialization or lateralization less than 10 mm. Lateral cup placement more than 10 mm in group B significantly exceeded that of group A. One possible explanation may be the higher frequency of Crowe III hips in group B, resulting in more cups placed in a higher position. Due to the funnel‐shaped geometry of the bony pelvis, it is more difficult for medialization when the center of rotation was elevated increasingly higher. Nevertheless, it should be stated that no complications such as loosening and liner wear occurred in our hips with excessive lateralization. In contrast to other studies which utilized metal on polyethylene bearing surfaces, we used a ceramic on ceramic (COC) interface in 91.8% of cases, as we hypothesized that the favorable wear characteristics of COC bearing surfaces may counteract the excessive joint reaction forces related to lateralization.

### *Effect of High Hip Center on the Abductor Strength and Postoperative Limp*

Some authors indicated that there is a negative correlation of abductor strength with a high rotation center of the hip. Through a radiological and biomechanical study, Abolghasemian *et al*.^25^ suggested that elevated hip center resulted in a decrease in the muscle length and a corresponding decrease in the preload, leading to weakness of abductor strength. But in a recent study, Traina *et al*.^26^ demonstrated that restoration of optimal femoral offset and abductor lever arm produced satisfactory results even for a center of hip rotation of >30 mm. We also reported low rates of limp and Trendelenburg sign in our HHC patients, although muscle strength was not quantitatively assessed. Though the height of hip center in group B significantly exceeded that in group A, the clinical and radiographic outcomes were similar after restoration of leg length, FO, and ALA, and no significant difference was shown in the two groups. In spite of the slack of gluteus medius due to elevated hip center, a larger size stem and appropriate head/neck lengths could be applied as compensation and could also contribute to correcting leg length discrepancy, avoiding limp of lower limbs. Further, preserving the continuity of abductors meant a favorable event regarding the restoration of normal gait. In our series, only 8.2% of all hips presented with a positive Trendelenburg sign and 8.7% of patients presented with a limp. The result of Trendelenburg sign was superior to the cases described by Chen *et al*.^21^ (14.2%) and Fukui *et al*.^10^ (13%).

### *Survival Rates of Implants at the Final Follow‐up*

In our series, the survival rates of implants at the final follow‐up were high: 96.7% (95%CI, 90.5%–100%) in group A and 96.2% (95%CI, 89.0%–100%) in group B. Comparison of our survivorships with other studies showed that the HHC technique was a reliable alternative method for Crowe II–III DDH.^7^, ^8^ Meanwhile, higher hip center did not significantly reduce the survivorship of implants at medium term even if it was above 28 mm.

### *Limitations of the Study*

This study has some limitations. First, our conclusion is based on a relatively small sample size. In addition, the validation of HHC technique needs a longer follow‐up. Second, this is a retrospective study. However, our patients were identified from a consecutive series with DDH, which may reduce the possibility of selection bias. Third, there is a lack of comparison between HHC technique and other methods. Fourth, the measures of gait used in this study were somewhat crude compared to other studies which undertook formal gait analysis. Furthermore, gluteus medius strength was not quantified because it was measured using a crude clinical test (Trendelenburg sign) instead of dynamometer machine. Thus, our results could only indicate that there appears to be enough strength in the abductors to avoid a Trendelenburg sign in the majority of cases. However, we believe that we have demonstrated good medium‐term results with a HHC technique which lends credibility to this technique and may serve as a benchmark for further research to assess longer‐term outcomes and to compare this technique with anatomic hip center techniques.

### *Conclusion*

We believed that HHC technique could be a valuable alternative in THA for Crowe II–III DDH. Further, between the groups with differing degrees of HHC, there were no significant differences in outcomes or survivorship in our study. However, larger comparative studies are required to confirm the implications of HHC THA definitively.

## Author Contributions

JMS: designing the study, analyzing the data, writing the manuscript; YGZ: designing the study, editing the manuscript; JYS, HYM, and YQD: collecting the data, analyzing the data, reviewing the manuscript; TJL: reviewing the literature. All authors have read and approved the final version of this manuscript.

## Authorship Declaration

All authors listed meet the authorship criteria according to the latest guidelines of the International Committee of Medical Journal Editors. All authors are in agreement with the manuscript.

## Ethics Approval and Consent to Participate

The Ethics Committee of our hospital, General Hospital of Chinese People's Liberation Army, approved the study protocol. A certificate of approval has been provided. The requirement of informed consent was exempted due to the retrospective nature of the study.
